# Intestinal antibody responses to a live oral poliovirus vaccine challenge among adults previously immunized with inactivated polio vaccine in Sweden

**DOI:** 10.1136/bmjgh-2019-001613

**Published:** 2019-08-28

**Authors:** Elizabeth B Brickley, Ruth I Connor, Wendy F Wieland-Alter, Marc S Collett, Marianne Hartford, Harrie Van Der Avoort, Austin W Boesch, Joshua A Weiner, Margaret E Ackerman, Mark A McKinlay, Minetaro Arita, Ananda S Bandyopadhyay, John F Modlin, Peter F Wright

**Affiliations:** 1 Infectious Disease Epidemiology, London School of Hygiene and Tropical Medicine, London, UK; 2 Epidemiology, Dartmouth College Geisel School of Medicine, Hanover, New Hampshire, USA; 3 Pediatrics, Dartmouth-Hitchcock Medical Center, Lebanon, New Hampshire, USA; 4 ViroDefense Inc, Chevy Chase, Maryland, USA; 5 Clinical Trial Center, Sahlgrenska University Hospital, Goteborg, Sweden; 6 Center for Infectious Disease Control, National Institute of Public Health and the Environment, Bilthoven, The Netherlands; 7 Thayer School of Engineering at Dartmouth, Hanover, New Hampshire, USA; 8 Task Force for Global Health, Decatur, Georgia, USA; 9 Department of Virology II, National Institute of Infectious Diseases, Shinjuku-ku, Tokyo, Japan; 10 Bill and Melinda Gates Foundation, Seattle, Washington, USA

**Keywords:** poliomyelitis, vaccines, immunisation, clinical trial

## Abstract

**Background:**

Our understanding of the acquisition of intestinal mucosal immunity and the control of poliovirus replication and transmission in later life is still emerging.

**Methods:**

As part of a 2011 randomised, blinded, placebo-controlled clinical trial of the experimental antiviral agent pocapavir (EudraCT 2011-004804-38), Swedish adults, aged 18–50 years, who had previously received four doses of inactivated polio vaccine (IPV) in childhood were challenged with a single dose of monovalent oral polio vaccine type 1 (mOPV1). Using faecal samples collected before and serially, over the course of 45 days, after mOPV1 challenge from a subset of placebo-arm participants who did not receive pocapavir (N=12), we investigated the kinetics of the intestinal antibody response to challenge virus by measuring poliovirus type 1-specific neutralising activity and IgA concentrations.

**Results:**

In faecal samples collected prior to mOPV1 challenge, we found no evidence of pre-existing intestinal neutralising antibodies to any of the three poliovirus serotypes. Despite persistent high-titered vaccine virus shedding and rising serum neutralisation responses after mOPV1 challenge, intestinal poliovirus type 1-specific neutralisation remained low with a titer of ≤18.4 across all time points and individuals. Poliovirus types 1-specific, 2-specific and 3-specific IgA remained below the limit of detection for all specimens collected postchallenge.

**Interpretation:**

In contrast to recent studies demonstrating brisk intestinal antibody responses to oral polio vaccine challenge in young children previously vaccinated with IPV, this investigation finds that adults previously vaccinated with IPV have only modest intestinal poliovirus type 1-specific neutralisation and no IgA responses that are measurable in stool samples following documented mOPV1 infection.

**Key questions**

**What is already known?**

- Although highly effective at protecting individuals from paralytic poliomyelitis, a childhood immunisation schedule based on inactivated polio vaccine (IPV) has a limited ability to inhibit intestinal viral replication on subsequent exposure to either live vaccine virus or circulating wild-type virus.
- Recent trials have demonstrated that infants challenged with live oral poliovirus vaccine following primary series with IPV rapidly develop intestinal poliovirus-specific neutralising antibody responses that are associated with reduced enteric viral replication. The impact of oral poliovirus vaccine challenge on intestinal poliovirus-specific neutralising antibody responses in adults is unknown.

**What are the new findings?**

- In contrast to studies conducted in infants, adults who received IPV in early childhood did not develop intestinal antibody responses on challenge with monovalent oral polio vaccine type 1.

**What do the new findings imply?**

- This study raises concern that adults are unlikely to mount intestinal antibody responses that protect against viral replication and shedding on exposure to live polio vaccine in later life.
- These findings imply that existing oral polio vaccines may be less effective at inducing transmission-blocking intestinal antibody responses in adults than they are in children.

## Introduction

To achieve global polio eradication, we must halt the transmission of all polioviruses. Vaccines that induce robust intestinal neutralising immune responses and interrupt poliovirus replication on mucosal surfaces continue to serve as the essential tools for realising this goal.^1 2^ Nevertheless, the magnitude of mucosal immunity that can be induced by vaccination is highly heterogeneous and can be modulated by the type and timing of the delivered vaccine schedule^3–5^ as well as recipient-specific characteristics, including factors related to the environment^6^ and enteric virome.^7 8^

Today, >50 years of scientific research confirm that live-attenuated oral polio vaccines (OPVs) administered in childhood are capable of stimulating the production of poliovirus-specific neutralising antibodies in nasopharyngeal and gastrointestinal mucosal tissues^9–12^ and thereby inhibiting poliovirus replication on subsequent homologous OPV challenge.^13–15^ In contrast, current evidence suggests childhood immunisation schedules based exclusively on inactivated (killed) polio vaccines (IPVs) induce only negligible intestinal immunity^3 9 11 16^ and fail to interrupt viral replication on subsequent exposure to either vaccine virus^14 17 18^ or circulating wild-type viruses.^19–21^

Potential interactions between OPV and IPV in the context of mucosal immunity remains an area of active inquiry. Several studies have suggested that paediatric OPV schedules (ie, OPV-first schedules) may educate the intestinal immune system such that a supplemental late dose of IPV may significantly boost children’s preexisting mucosal neutralising activity.^5 22 23^ On the other hand, a series of recent trials in Latin American infants have demonstrated that children who instead received primary vaccine series with IPV (ie, IPV-first schedules) shed high quantities of vaccine virus after receiving a supplemental challenge dose of OPV,^17 18^ but consistently developed strong poliovirus type-specific enteric neutralising activity by 2 weeks’ post-OPV challenge.^11 12^

We hypothesised that, like their paediatric counterparts, adults whose only known poliovirus experience was IPV receipt in early childhood would have limited intestinal immunity at baseline, but would develop robust intestinal antibody responses when challenged with a live OPV. As part of a 2011 randomised clinical trial of the experimental antiviral agent pocapavir, Swedish adults who had exclusively received IPV in childhood were challenged with a single dose of monovalent oral polio vaccine type 1 (mOPV1). The primary results of the trial by Collett and colleagues demonstrate an antiviral effect of pocapavir, but also described mOPV1 shedding in the placebo-arm participants (N=48) with a median duration of virus excretion of 13 days.^24^ To investigate the impact of this sustained viral replication on the acquisition of mucosal immunity in adults, we assayed faecal samples collected serially after mOPV1 challenge from a randomly selected subset of the placebo-arm participants. The kinetics of the type 1-specific intestinal antibody response to mOPV1 challenge virus in adults were evaluated by measuring poliovirus type-specific neutralising activity and IgA concentrations in stool samples.

## Methods

### Study design and participants

The design and primary outcomes of the phase I, randomised, blinded, placebo-controlled trial undertaken between 10 January and 18 December 2012 have been described previously.^24^ The study protocol is available at: https://www.clinicaltrialsregister.eu/ctr-search/trial/2011-004804-38/SE. Trial participants included healthy Swedish volunteers, aged 18–50 years, who had received the recommended Swedish childhood vaccination schedule of four IPV injections.^24^ Individuals were screened for prior exposure to live poliovirus, and individuals both positive for total serum IgA and negative for poliovirus-specific serum IgA were eligible for participation.

For the trial, all participants were challenged with a single dose of mOPV1 (median cell culture infective dose (CCID_50_), 10^6^) and then followed for 45 days. Faecal specimens were collected from all participants prior to the mOPV1 challenge and serially thereafter. Blood samples were collected at baseline and on the last day of the study. In the current analyses, a subset of twelve participants were selected at random from the 48 placebo-arm controls for further analysis of the intestinal antibody response. Depending on the trial arm, subjects received either a standard (fat content, <25 g) or a high-fat meal (fat content, 60–75 g) over the course of the 14-day placebo administration period.

### Laboratory procedures

Stool samples collected from the study participants and stored frozen at the National Institute for Public Health and the Environment (Bilthoven, The Netherlands) were identified, thawed, aliquoted and refrozen prior to shipping to the USA.^10 11 15 25^ This excluded any possibility of chloroform treatment, which is used in virus titration but has been previously shown to inactivate antibody activity (Wright laboratory, unpublished data). At Dartmouth Geisel School of Medicine (Hanover, New Hampshire, USA), investigators evaluated intestinal mucosal immunity by measuring poliovirus-specific neutralising activity and IgA in stool samples. As previously described, poliovirus type-specific neutralising activity was determined by limiting dilution inhibition of luciferase-expressing wild type-derived polio pseudoviruses in vitro and presented as the reciprocal of the highest sample dilution needed to achieve 60% neutralisation as compared with control wells with no added sample.^25^ Poliovirus-specific stool neutralisation titers of less than two were considered undetectable and recorded as one. In an effort to determine if any other pathogen-specific mucosal antibodies could be quantified in the stool specimens of these adults, investigators also measured intestinal neutralising activity to respiratory syncytial virus (RSV) and A/California/04/2009 H1N1 influenza using luciferase-based assays, similar to that used for poliovirus, in the stool samples collected 45 days’ postchallenge. Specifically, stool extracts were tested for RSV neutralisation in microtiter assays using a recombinant RSV-Renilla luciferase (rA2-Rluc) virus in HEp-2 target cells and based on previously published methods.^26^ Neutralisation of influenza was evaluated in Madin-Darby Canine Kidney (MDCK NBL-2) cells infected with A/California/04/2009 H1N1 virus (CA/09) encoding NanoLuc (NLuc) luciferase as described.^27^ Samples were serially diluted twofold prior to incubation with virus and then added to target cells. Luciferase expression was quantified in cell lysates after 24 hours at 37°C, using either the Renilla or NanoGlo Luciferase assay systems (Promega, Madison, Wisconsin, USA) for RSV and influenza, respectively. Relative light units were measured on a BioTek Synergy 2 microplate reader (Winooski, Vermont, USA). Neutralisation was again calculated as the reciprocal of the highest sample dilution to yield a 60% reduction in relative light units. Total and poliovirus type-specific concentrations of IgA in stool specimens were quantified, relative to a serum standard, using a multiplex Luminex-based microsphere assay developed by coupling monovalent IPVs to fluorescently coded magnetic microspheres.^15^ As described in the parent study, the quantification of type-specific serum antibody titers and mOPV1 viral shedding was performed at the National Institute for Public Health and the Environment using standard methods.^5 24^ Serum neutralisation titers less than eight were recorded as four.

### Statistical analysis

Longitudinal patterns in shedding and immune markers were evaluated by plotting viral shedding titers and poliovirus type-specific serum IgA and stool neutralisation titers by the days since mOPV1 challenge. Wilcoxon signed rank tests were used to compare type 1-specific serum neutralisation prechallenge and postchallenge. Distributions of virus-specific IgA in stool samples 45 days’ postchallenge were compared using column scatter graphs. All p values are from two-sided statistical tests, and all analyses were performed using Stata V.15.0 and R V.3.2.5.

### Patient and public involvement

Patients and the public were not involved in our work.

### Ethics and role of the funding source

The study was approved by the Göteborg Regional Ethical Review Board (2011-004804-38; 151:2012/62373) and was conducted in accordance with the Declaration of Helsinki, the International Conference on Harmonisation guidelines for Good Clinical Practice, and the codes and regulation of the USA and Sweden regarding research on human subjects. ASB and JFM are employees of the study funder and were involved in study design, data interpretation and writing of the report. The funder had no role in data collection. All authors had full access to the data in the study and share final responsibility for the decision to submit for publication.

## Results

Poliovirus type-specific intestinal antibody responses were evaluated in 156 stool samples from 12 adult placebo-arm subjects participating in the mOPV1 challenge trial. Total IgA was successfully detected in stool samples from all 12 participants. The median concentration of total IgA at baseline was measured to be 28 000 ng/mL (IQR: 14 000–63 000). Pre-existing intestinal neutralising antibody titers specific to the three poliovirus serotypes were all measured to be ≤3.3 in the stool samples collected prior to challenge, and poliovirus types 1-specific, 2-specific and 3-specific stool IgA levels were undetectable for all participants at baseline.

**Figure 1:**
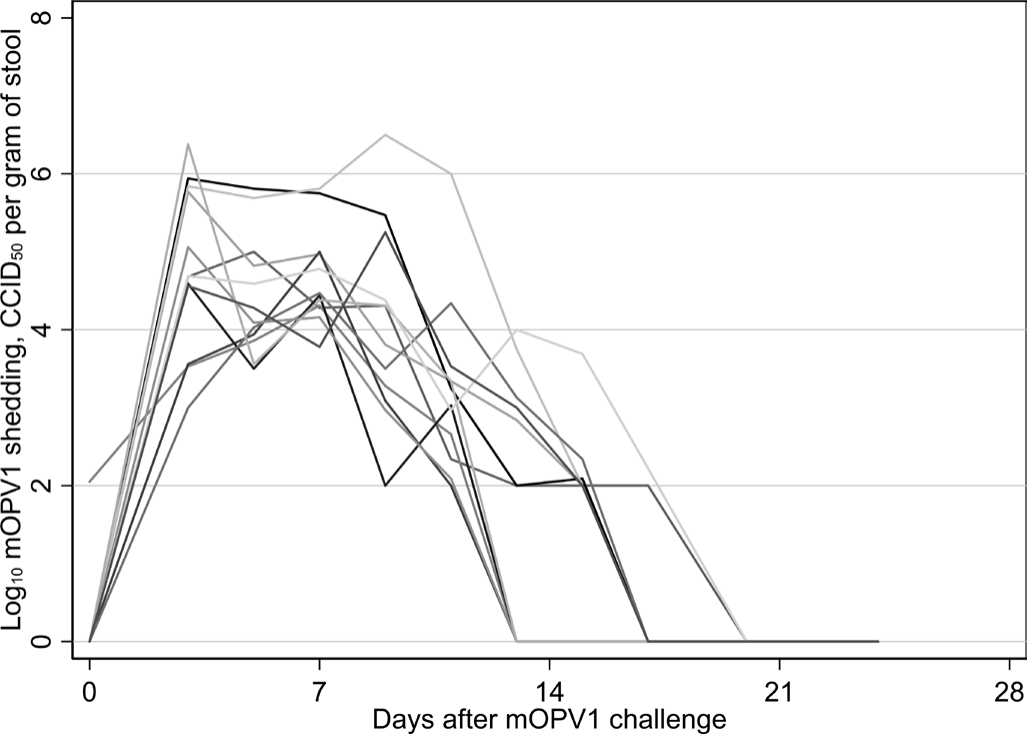
Monovalent oral polio vaccine type 1 viral shedding (log_10_ CCID_50_ per gram of stool) in the 4 weeks following challenge among the 12 placebo-arm participants in the study sample.

**Figure 2:**
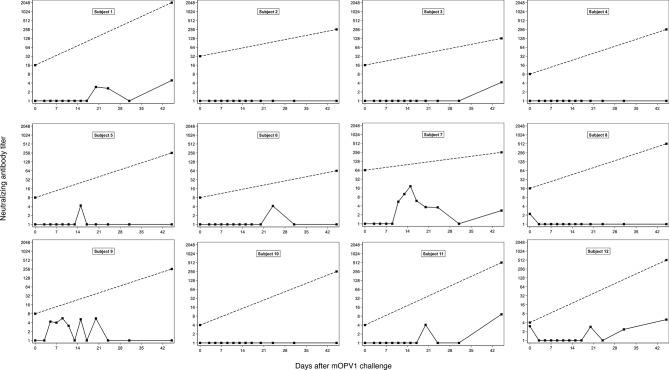
Subject-specific poliovirus type 1-specific serum IgA (dashed line) and stool neutralising antibody titers (solid line) at the time of mOPV1 challenge and in the 45 days after challenge in adults previously vaccinated with four doses of IPV in childhood (N=12). mOPV1, monovalent oral polio vaccine type 1.

**Figure 3:**
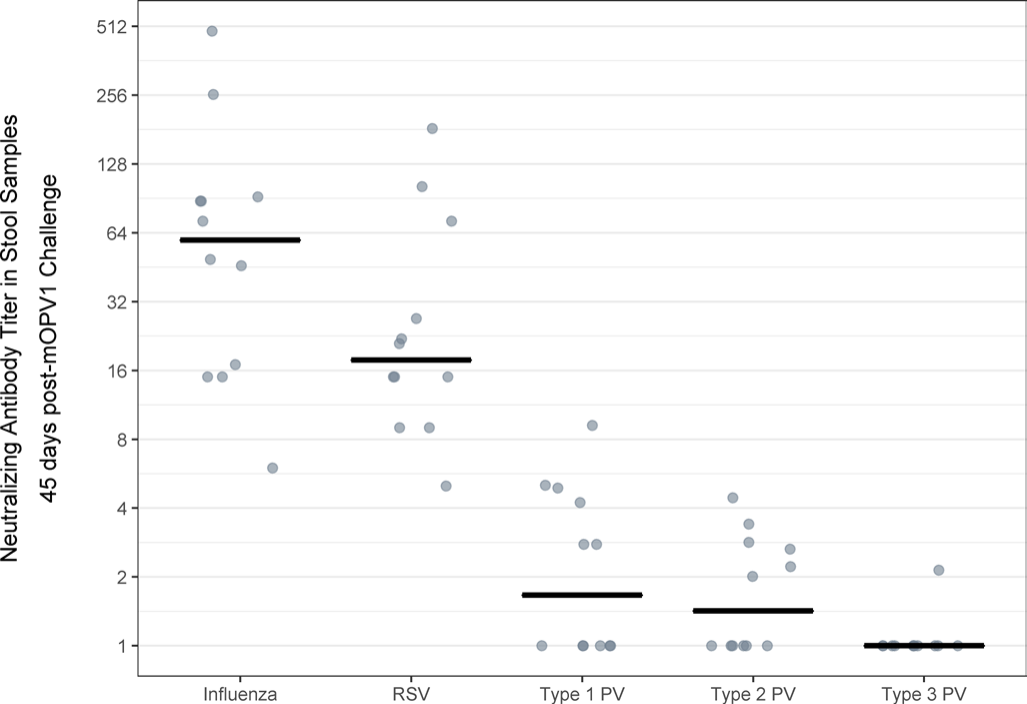
Neutralising antibody titers for A/California/04/2009 H1N1 influenza, respiratory syncytial virus (RSV) and poliovirus (PV) types 1, 2 and 3 in stool samples collected 45 days’ postchallenge with monovalent oral polio vaccine type 1 in adults previously vaccinated with four doses of IPV in early childhood (N=12).

Following challenge, the 12 subjects experienced sustained viral shedding that lasted for between 11 and 17 days and reached a median peak log_10_ viral shedding titer of 5.0 CCID_50_ per gram of stool (IQR: 4.7–5.9) (figure 1). Despite substantial virus shedding, no corresponding intestinal IgA or neutralisation responses were observed. In the stool samples collected in the 6 weeks after challenge, poliovirus types 1-specific, 2-specific and 3-specific IgA remained below the limit of detection for all 141 tested specimens. Similarly, poliovirus type 1-specific stool neutralising antibody titers remained low (ie, ≤18.4 across all time points and individuals) (figure 2). Likewise, poliovirus types 2-specific and 3-specific neutralising antibody titers in the stool samples showed no substantive changes postchallenge (ie, titers remained at ≤7.2 for type 2 and ≤21.2 for type 3 across all time points and individuals) (online supplementary file 1). In contrast, poliovirus type 1-specific serum neutralising activity increased significantly after OPV1 challenge (p=0.002, Wilcoxon signed rank test), with a median fold-change of 32 (IQR: 8–96) and a median day-45 serum neutralising antibody titer of 256 (IQR: 256–512) (figure 2). Further, whereas intestinal antibodies to all three types of poliovirus exhibited low neutralising activity in stool samples at 45 days’ postchallenge (median, IQR for type 1: 1, 1–4.6; type 2: 1.5, 1–2.7; type 3: 1, 1–1), neutralising antibodies were detectable in the stool samples for both RSV (median, IQR: 21.5, 15–50) and A/California/04/2009 H1N1 influenza virus (median, IQR: 74, 31.5–90) (figure 3). The type of diet received did not alter any immunologic parameter measured.

10.1136/bmjgh-2019-001613.supp1 Supplementary data

## Discussion

With the declining circulation of wild polioviruses worldwide, health systems are becoming increasingly reliant on IPV as the principal tool for achieving global polio eradication. Though it is well established that IPV-driven schedules are highly effective at minimising paralytic polio risks across the lifespan, the limited capacity of IPV schedules delivered in childhood to confer intestinal immunity capable of interrupting poliovirus transmission remains an important consideration for the global polio eradication strategy.^28^ Using samples collected from a subset of 12 placebo-arm participants administered mOPV1 in a randomised controlled trial of a potential polio antiviral, pocapavir,^24^ we investigated intestinal immunity to poliovirus in adult participants by measuring poliovirus type-specific neutralising activity and IgA responses in faecal samples. In samples collected at baseline (ie, prior to mOPV1 challenge), we found no evidence of pre-existing intestinal immunity to any of the three poliovirus serotypes in this group of Swedish adults with a prior history of IPV immunisation in childhood. Surprisingly, we detected only minimal type 1-specific stool neutralisation and no stool IgA responses following live OPV challenge. Assuming that the assay performance in adult stool samples is robust, the results raise concerns about the effectiveness of OPV vaccination in boosting mucosal immunity among older individuals and, therefore, have potentially important implications for the planning of vaccine provisioning as part of the Global Polio Eradication Initiative’s outbreak control activities.

Our finding that IPV receipt in childhood was not associated with detectable prechallenge levels of intestinal poliovirus-specific neutralising activity and IgA in adulthood aligns with prior OPV challenge studies as well as observational epidemiologic evidence. As part of a 1990 American clinical trial in which children were vaccinated with an enhanced-potency IPV at 2, 4 and 18 months of age prior to challenge with mOPV1, 68% of children had undetectable poliovirus type 1-specific stool IgA following the primary IPV vaccine series, while 63% went on to shed vaccine virus on challenge.^29^ Similarly, in a 2010 Omani clinical trial in which infants were vaccinated with IPV at 2, 4 and 6 months and then challenged with mOPV1 at 7 months, 73% of the infants had undetectable (ie, <2) poliovirus type 1-specific stool neutralising titers following the primary IPV vaccine series,^15^ while 63% went on to shed vaccine virus on challenge.^30^ Analogous failures to induce poliovirus type 2-specific mucosal immunity by IPV have been observed as part of trials in Panama^11^ and Chile^12^ among infants who were vaccinated with three doses of IPV before challenge with mOPV2. Furthermore, in a faecal monitoring study conducted in response to the 2013 silent outbreak of wild poliovirus type 1 in Israel, 85% of faecal samples that were positive for virus were collected from children (<10 years of age) who had been previously vaccinated with at least three doses of IPV.^31^

The observation that, despite documented viral replication and sustained excretion, the Swedish study participants failed to mount an intestinal antibody response to mOPV1 challenge was unanticipated and in marked contrast to the experiences of five OPV challenge studies in paediatric participants.^10–12 15 29^ For both of the aforementioned mOPV1 challenge studies in the USA^29^ and Oman,^15^ rises in poliovirus type 1-specific intestinal immune markers (ie, IgA in stool specimens and neutralising activity, respectively) were reported in the IPV recipients following mOPV1 receipt. Further, in the mOPV2 challenge studies in Panama^11^ and Chile,^12^ the poliovirus type 2-specific intestinal antibody responses appeared similar to the poliovirus type 1-specific antibody responses in the American and Omani studies and different from that observed in the current study. Specifically, infants with detectable viral shedding following mOPV2 challenge exhibited brisk intestinal antibody responses, with significant increases in poliovirus type 2-specific stool IgA and neutralising activity titers by 2 weeks’ postchallenge. Parallel rises in poliovirus type 2-specific mucosal immune markers following mOPV2 challenge have also been reported among infants from Guatemala and the Dominican Republic who received a primary immunisation series of three doses of bivalent OPV (ie, targeting poliovirus types 1 and 3) plus one dose of IPV.^10^

Although the finding of an absence of an intestinal antibody response to mOPV1 challenge in adults was unexpected, there is some indirect evidence in the literature of an age-related diminution of intestinal immunity to poliovirus. Surveillance data of viral excretion in Indian children under age 5 indicate that intestinal immunity in children vaccinated with OPV wanes within the first year after OPV exposure.^32^ Consistent with this model, an early report of British young men aged 16–18 years found that 20% of the participants who had been previously vaccinated with OPV (ie, on average 5 years prior) excreted poliovirus type 1 on subsequent trivalent OPV challenge.^33^ Similarly, in a study of the induction of mucosal immunity by IPV boosting in adults aged 20–44 years, investigators reported detecting poliovirus types 1-specific, 2-specific and 3-specific IgA in stool samples from only three of nine adults who had received a primary vaccine series of OPV in childhood.^5^

We also found complementary evidence for the biological plausibility of the observed phenomenon through further testing of available study samples. First, we observed that all 12 participants mounted serum neutralisation responses. Second, we were able to detect total IgA successfully in stool samples from each of the participants. Although the total quantified concentrations of intestinal IgA in the adults were lower than that previously reported in studies of breastfed infants,^12^ the concentrations were similar to those observed among infants with mixed or formula feeding (data not shown). Third, although we considered other hypothetical explanations relating to the induction of a tolerance or senescence of the mucosal immune system in adulthood, we observed that the participants’ stool samples had neutralising activity against two other commonly circulating viruses, RSV and influenza.

Mucosal immunity to RSV and influenza has been demonstrated in the setting of natural infection and in response to immunisation with live and inactivated vaccines.^34–37^ Neutralisation of these viruses has been assessed in the past using mucosal specimens, including nasal washes, collected from the upper respiratory tract. Our data are the first to demonstrate neutralisation of RSV and influenza by samples from the gastrointestinal tract (eg, stool extracts). While our observations are preliminary and will require further study, this finding is consistent with the broad concept of a common mucosal immune system and the induction of virus-specific immunity at sites distal from the location of primary infection.

Despite this being the first OPV challenge study that directly measured intestinal antibody responses to poliovirus strains in adult populations, this investigation has limitations. By design, the mOPV1 challenge trial restricted enrollment to immune-competent individuals who were negative for serum poliovirus-specific IgA at baseline (as a control for potential past exposure to live poliovirus). While this selection criterion maximised the likelihood of mOPV1 replication on challenge, it limits the generalisability of our findings across the general population of adults and also potentially biases the study towards subjects who may have been incapable of mounting intestinal antibody responses to poliovirus. Notably, in contrast to the prior OPV challenge studies of IPV recipients, the setting in Sweden is unique in that routine childhood vaccination was historically done with IPV. The findings of this study, while consistent across all participants, are also constrained by the small number of study subjects.

Looking to the future, it will be valuable to validate these findings across different age ranges (ie, in cohorts of older children, adolescents and adults) and to explore whether there could be any age-mediated restrictions to the amount of secreted IgA that is detectable in stool. The investigation should also be replicated across older individuals with different vaccine histories (ie, in participants who received OPV vaccination schedules in childhood) to determine whether the observed deficit in response could be influenced by prior vaccine experience. Further, as the primary site of poliovirus replication and immune response is in the small intestine, we have considered that IgA may be degraded by proteases while in transit through the adult intestinal tract, and as a result, the observation of a lack of poliovirus-specific intestinal antibody response is the subject of on-going experimentation in our laboratory. Further, it will be important to explore additional immunological responses (eg, cell-mediated immunity) or body fluids (eg, saliva), which may contribute to the participants’ ability to control the challenge virus infections in the absence of a detectable antibody-mediated response in the intestinal mucosae.

In conclusion, the reported failure to detect an intestinal antibody response to mOPV1 challenge in an adult population is intriguing and evokes questions about the nature and regulation of the mucosal immune response to serial poliovirus vaccination. These findings imply that existing OPVs may be less effective at inducing transmission-blocking intestinal antibody responses in IPV-vaccinated adults than they are in children. The findings also highlight the importance of considering the impact of existing and new polio vaccines (eg, novel live-attenuated OPVs)^38^ in terms of their ability to elicit mucosal immunity and limit onwards transmission across a wide range of subjects.

## References

[1] Bandyopadhyay AS, Garon J, Seib K, et al Polio vaccination: past, present and future. Future Microbiol 2015;10:791–808. 10.2217/fmb.15.19 25824845

[2] Sutter RW, Platt L, Mach O, et al The new polio eradication end game: rationale and supporting evidence. J Infect Dis 2014;210(suppl 1):S434–8. 10.1093/infdis/jiu222 25316865

[3] Parker EP, Molodecky NA, Pons-Salort M, et al Impact of inactivated poliovirus vaccine on mucosal immunity: implications for the polio eradication endgame. Expert Rev Vaccines 2015;14:1113–23. 10.1586/14760584.2015.1052800 26159938 PMC4673562

[4] Modlin JF, Halsey NA, Thoms ML, et al Humoral and mucosal immunity in infants induced by three sequential inactivated poliovirus vaccine-live attenuated oral poliovirus vaccine immunization schedules. Baltimore area polio vaccine Study Group. J Infect Dis 1997;175 Suppl 1(Supplement 1):S228–S234. 10.1093/infdis/175.Supplement_1.S228 9203721

[5] Herremans TM, Reimerink JH, Buisman AM, et al Induction of mucosal immunity by inactivated poliovirus vaccine is dependent on previous mucosal contact with live virus. J Immunol 1999;162:5011–8. 10202050

[6] Grassly NC, Jafari H, Bahl S, et al Mucosal immunity after vaccination with monovalent and trivalent oral poliovirus vaccine in India. J Infect Dis 2009;200:794–801. 10.1086/605330 19624278

[7] Praharaj I, Parker EPK, Giri S, et al Influence of non-polio enteroviruses and the bacterial gut microbiota on oral poliovirus vaccine response: a study from South India. J Infect Dis 2018. 10.1093/infdis/jiy568 PMC6601701 30247561

[8] Parker EPK, Kampmann B, Kang G, et al Influence of enteric infections on response to oral poliovirus vaccine: a systematic review and meta-analysis. J Infect Dis 2014;210:853–64. 10.1093/infdis/jiu182 24688069 PMC4136801

[9] Ogra PL, Karzon DT, Righthand F, et al Immunoglobulin response in serum and secretions after immunization with live and inactivated poliovaccine and natural infection. N Engl J Med 1968;279:893–900. 10.1056/NEJM196810242791701 20617594

[10] Wright PF, Connor RI, Wieland-Alter WF, et al Vaccine-Induced mucosal immunity to poliovirus: analysis of cohorts from an open-label, randomised controlled trial in Latin American infants. Lancet Infect Dis 2016;16:1377–84. 10.1016/S1473-3099(16)30169-4 27638357 PMC5611465

[11] Brickley EB, Strauch CB, Wieland-Alter WF, et al Intestinal immune responses to type 2 oral polio vaccine (OPV) challenge in infants previously immunized with bivalent OPV and either high-dose or standard inactivated polio vaccine. J Infect Dis 2018;217:371–80. 10.1093/infdis/jix556 29304199 PMC5853416

[12] Brickley EB, Wieland-Alter W, Connor RI, et al Intestinal immunity to poliovirus following sequential trivalent inactivated polio Vaccine/Bivalent oral polio vaccine and trivalent inactivated polio Vaccine-only immunization schedules: analysis of an open-label, randomized, controlled trial in Chilean infants. Clin Infect Dis 2018;67(suppl_1):S42–50. 10.1093/cid/ciy603 30376086 PMC6206105

[13] Sabin AB, Michaels RH, Ziring P, et al Effect of oral poliovirus vaccine in newborn children. II. intestinal resistance and antibody response at 6 months in children fed type I vaccine at birth. Pediatrics 1963;31:641–50. 13975877

[14] Hird TR, Grassly NC Systematic review of mucosal immunity induced by oral and inactivated poliovirus vaccines against virus shedding following oral poliovirus challenge. PLoS Pathog 2012;8:e1002599 10.1371/journal.ppat.1002599 22532797 PMC3330118

[15] Wright PF, Wieland-Alter W, Ilyushina NA, et al Intestinal immunity is a determinant of clearance of poliovirus after oral vaccination. J Infect Dis 2014;209:1628–34. 10.1093/infdis/jit671 24459191

[16] Modlin JF Mucosal immunity following oral poliovirus vaccine and enhanced potency inactivated poliovirus vaccine immunization. Pediatr Infect Dis 1991;10:976–8. 10.1097/00006454-199112000-00031 1766726

[17] O'Ryan M, Bandyopadhyay AS, Villena R, et al Inactivated poliovirus vaccine given alone or in a sequential schedule with bivalent oral poliovirus vaccine in Chilean infants: a randomised, controlled, open-label, phase 4, non-inferiority study. Lancet Infect Dis 2015;15:1273–82. 10.1016/S1473-3099(15)00219-4 26318714

[18] Sáez-Llorens X, Clemens R, Leroux-Roels G, et al Immunogenicity and safety of a novel monovalent high-dose inactivated poliovirus type 2 vaccine in infants: a comparative, observer-blind, randomised, controlled trial. Lancet Infect Dis 2016;16:321–30. 10.1016/S1473-3099(15)00488-0 26719058 PMC9108810

[19] Shulman LM, Gavrilin E, Jorba J, et al Molecular epidemiology of silent introduction and sustained transmission of wild poliovirus type 1, Israel, 2013. Euro Surveill 2014;19 10.2807/1560-7917.ES2014.19.7.20709 24576471

[20] Shulman LM, Martin J, Sofer D, et al Genetic analysis and characterization of wild poliovirus type 1 during sustained transmission in a population with >95% vaccine coverage, Israel 2013. Clin Infect Dis 2015;60:1057–64. 10.1093/cid/ciu1136 25550350

[21] Schaap GJ, Bijkerk H, Coutinho RA, et al The spread of wild poliovirus in the well-vaccinated Netherlands in connection with the 1978 epidemic. Prog Med Virol 1984;29:124–40. 6322230

[22] John J, Giri S, Karthikeyan AS, et al The duration of intestinal immunity after an inactivated poliovirus vaccine booster dose in children immunized with oral vaccine: a randomized controlled trial. J Infect Dis 2017;215:529–36. 10.1093/infdis/jiw595 28003352 PMC5388294

[23] Jafari H, Deshpande JM, Sutter RW, et al Polio eradication. efficacy of inactivated poliovirus vaccine in India. Science 2014;345:922–5. 10.1126/science.1255006 25146288 PMC10389671

[24] Collett MS, Hincks JR, Benschop K, et al Antiviral activity of Pocapavir in a randomized, blinded, placebo-controlled human oral poliovirus vaccine challenge model. J Infect Dis 2017;215:335–43. 10.1093/infdis/jiw542 27932608 PMC5393058

[25] Arita M, Iwai M, Wakita T, et al Development of a poliovirus neutralization test with poliovirus pseudovirus for measurement of neutralizing antibody titer in human serum. Clin Vaccine Immunol 2011;18:1889–94. 10.1128/CVI.05225-11 21880850 PMC3209023

[26] Fuentes S, Crim RL, Beeler J, et al Development of a simple, rapid, sensitive, high-throughput luciferase reporter based microneutralization test for measurement of virus neutralizing antibodies following respiratory syncytial virus vaccination and infection. Vaccine 2013;31:3987–94. 10.1016/j.vaccine.2013.05.088 23742994 PMC3779065

[27] Tran V, Moser LA, Poole DS, et al Highly sensitive real-time in vivo imaging of an influenza reporter virus reveals dynamics of replication and spread. J Virol 2013;87:13321–9. 10.1128/JVI.02381-13 24089552 PMC3838222

[28] Brickley EB, Wright PF Maximising the impact of inactivated polio vaccines. Lancet Infect Dis 2017;17:680–1. 10.1016/S1473-3099(17)30236-0 28454675 PMC6047357

[29] Onorato IM, Modlin JF, McBean AM, et al Mucosal immunity induced by enhance-potency inactivated and oral polio vaccines. J Infect Dis 1991;163:1–6. 10.1093/infdis/163.1.1 1845806

[30] Mohammed AJ, AlAwaidy S, Bawikar S, et al Fractional doses of inactivated poliovirus vaccine in Oman. N Engl J Med 2010;362:2351–9. 10.1056/NEJMoa0909383 20573923

[31] Moran-Gilad J, Mendelson E, Burns CC, et al Field study of fecal excretion as a decision support tool in response to silent reintroduction of wild-type poliovirus 1 into Israel. J Clin Virol 2015;66:51–5. 10.1016/j.jcv.2015.03.005 25866337

[32] Grassly NC, Jafari H, Bahl S, et al Waning intestinal immunity after vaccination with oral poliovirus vaccines in India. J Infect Dis 2012;205:1554–61. 10.1093/infdis/jis241 22448007

[33] Smith JW, Lee JA, Fletcher WB, et al The response to oral poliovaccine in persons aged 16-18 years. J Hyg 1976;76:235–47. 10.1017/S0022172400055133 177700 PMC2129633

[34] Bagga B, Cehelsky JE, Vaishnaw A, et al Effect of preexisting serum and mucosal antibody on experimental respiratory syncytial virus (RSV) challenge and infection of adults. J Infect Dis 2015;212:1719–25. 10.1093/infdis/jiv281 25977264

[35] Etchart N, Baaten B, Andersen SR, et al Intranasal immunisation with inactivated RSV and bacterial adjuvants induces mucosal protection and abrogates eosinophilia upon challenge. Eur J Immunol 2006;36:1136–44. 10.1002/eji.200535493 16619288

[36] Wright PF, Hoen AG, Ilyushina NA, et al Correlates of immunity to influenza as determined by challenge of children with live, attenuated influenza vaccine. Open Forum Infect Dis 2016;3 10.1093/ofid/ofw108 PMC4943547 27419180

[37] Brickley EB, Wright PF, Khalenkov A, et al The effect of preexisting immunity on virus detection and immune responses in a phase II, randomized trial of a Russian-Backbone, live, attenuated influenza vaccine in Bangladeshi children. Clin Infect Dis 2018;35 10.1093/cid/ciy1004 PMC6695513 30481269

[38] Van Damme P, De Coster I, Bandyopadhyay AS, et al The safety and immunogenicity of two novel live attenuated monovalent (serotype 2) oral poliovirus vaccines in healthy adults: a double-blind, single-centre phase 1 study. The Lancet 2019;394:148–58. 10.1016/S0140-6736(19)31279-6 PMC6626986 31174831

