# Supplementary material for: Intestinal antibody responses to a live oral poliovirus vaccine challenge among adults previously immunized with inactivated polio vaccine in Sweden
Source: BMJ Glob Health. 2019 Aug 28;4(4):e001613. doi: 10.1136/bmjgh-2019-001613 (PMC6730592; doi:10.1136/bmjgh-2019-001613)
Supplement: Supplementary data [file bmjgh-2019-001613supp001.pdf]

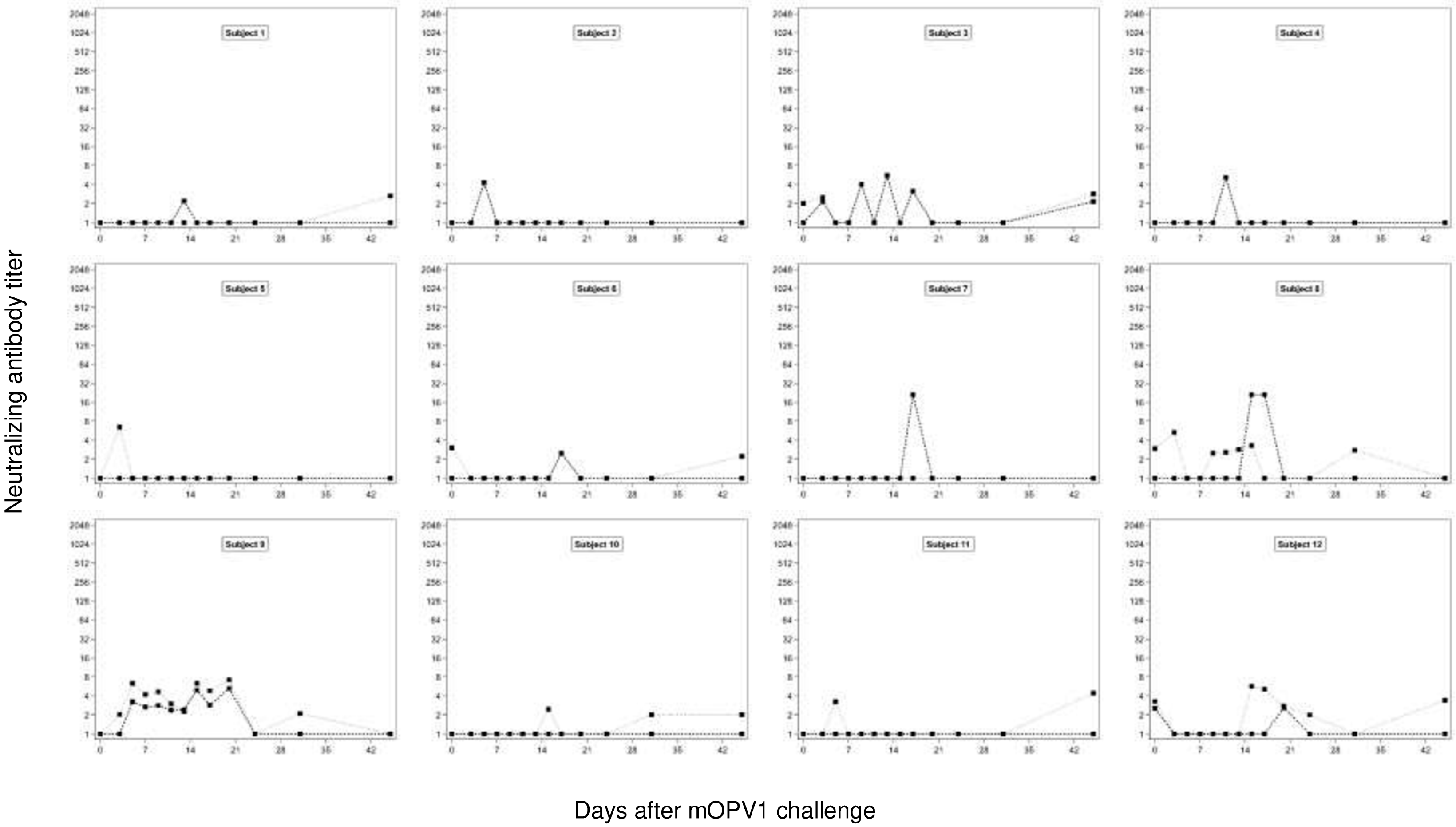

Supplementary Figure 1. Subject-specific neutralizing antibody titers in stool samples to poliovirus type 2 (dotted line) and type 3 (dashed line) at the time of mOPV1 challenge and in the 45 days after challenge in adults previously vaccinated with four doses of IPV in childhood (N=12). Squares indicate the days of specimen testing. Subjects 1-6, high fat diet; subjects 7-12, standard diet.
